# Supplementary material for: Modeling glioblastoma heterogeneity as a dynamic network of cell states
Source: Mol Syst Biol. 2021 Sep 16;17(9):e10105. doi: 10.15252/msb.202010105 (PMC8444284; doi:10.15252/msb.202010105)
Supplement: Supplementary file 5 — Source Data for Figure 3 [file MSB-17-e10105-s001.zip › Figure3A_sourcedata/GSEA_3065/nosigdb_state1.GseaPreranked.1622718409535/LARSSON_STATE_3.html]

Details for gene set LARSSON\_STATE\_3[GSEA]

|  || Dataset | state1 |
| Phenotype | NoPhenotypeAvailable |
| Upregulated in class | na\_neg |
| GeneSet | LARSSON\_STATE\_3 |
| Enrichment Score (ES) | -0.8618143 |
| Normalized Enrichment Score (NES) | -2.9912896 |
| Nominal p-value | 0.0 |
| FDR q-value | 0.0 |
| FWER p-Value | 0.0 |
Table: GSEA Results Summary

  

Fig 1: Enrichment plot: LARSSON\_STATE\_3      
 Profile of the Running ES Score & Positions of GeneSet Members on the Rank Ordered List

  

| PROBE | GENE SYMBOL | GENE\_TITLE | RANK IN GENE LIST | RANK METRIC SCORE | RUNNING ES | CORE ENRICHMENT || 1 | POSTN |  |  | 11 | 0.689 | 0.0216 | No |
| 2 | HSP90AB1 |  |  | 521 | 0.201 | -0.0238 | No |
| 3 | TIMP1 |  |  | 2497 | 0.040 | -0.2241 | No |
| 4 | HSP90AA1 |  |  | 2562 | 0.038 | -0.2294 | No |
| 5 | IQGAP1 |  |  | 2622 | 0.035 | -0.2343 | No |
| 6 | MKI67 |  |  | 2760 | 0.031 | -0.2472 | No |
| 7 | DST |  |  | 3373 | 0.017 | -0.3092 | No |
| 8 | CENPF |  |  | 6902 | -0.047 | -0.6678 | No |
| 9 | LAMC1 |  |  | 6974 | -0.049 | -0.6734 | No |
| 10 | CD320 |  |  | 7132 | -0.054 | -0.6877 | No |
| 11 | TMED9 |  |  | 7297 | -0.058 | -0.7025 | No |
| 12 | EMP3 |  |  | 7404 | -0.061 | -0.7114 | No |
| 13 | TOP2A |  |  | 7678 | -0.069 | -0.7370 | No |
| 14 | ASPM |  |  | 7821 | -0.074 | -0.7490 | No |
| 15 | TFPI2 |  |  | 8026 | -0.082 | -0.7671 | No |
| 16 | PLD5 |  |  | 8327 | -0.095 | -0.7946 | No |
| 17 | BSG |  |  | 8399 | -0.099 | -0.7986 | No |
| 18 | HNRNPU |  |  | 8616 | -0.112 | -0.8170 | No |
| 19 | GGH |  |  | 8643 | -0.114 | -0.8159 | No |
| 20 | LMAN1 |  |  | 9094 | -0.151 | -0.8568 | Yes |
| 21 | MANF |  |  | 9095 | -0.152 | -0.8518 | Yes |
| 22 | CD63 |  |  | 9126 | -0.154 | -0.8498 | Yes |
| 23 | TM9SF3 |  |  | 9212 | -0.167 | -0.8530 | Yes |
| 24 | MYH10 |  |  | 9244 | -0.172 | -0.8505 | Yes |
| 25 | PIF1 |  |  | 9278 | -0.176 | -0.8480 | Yes |
| 26 | FAT1 |  |  | 9301 | -0.181 | -0.8443 | Yes |
| 27 | SYNE2 |  |  | 9362 | -0.193 | -0.8441 | Yes |
| 28 | HSP90B1 |  |  | 9365 | -0.193 | -0.8379 | Yes |
| 29 | EMC7 |  |  | 9376 | -0.195 | -0.8325 | Yes |
| 30 | STT3B |  |  | 9414 | -0.204 | -0.8296 | Yes |
| 31 | CALU |  |  | 9418 | -0.205 | -0.8231 | Yes |
| 32 | NASP |  |  | 9437 | -0.208 | -0.8181 | Yes |
| 33 | SSR4 |  |  | 9449 | -0.213 | -0.8122 | Yes |
| 34 | PRDX4 |  |  | 9457 | -0.215 | -0.8058 | Yes |
| 35 | ITM2B |  |  | 9486 | -0.223 | -0.8014 | Yes |
| 36 | RPN2 |  |  | 9489 | -0.224 | -0.7942 | Yes |
| 37 | HSPA5 |  |  | 9533 | -0.234 | -0.7909 | Yes |
| 38 | CALR |  |  | 9537 | -0.235 | -0.7834 | Yes |
| 39 | PRKDC |  |  | 9539 | -0.236 | -0.7758 | Yes |
| 40 | SPTBN1 |  |  | 9608 | -0.263 | -0.7741 | Yes |
| 41 | SPCS3 |  |  | 9609 | -0.263 | -0.7654 | Yes |
| 42 | DYNC1H1 |  |  | 9632 | -0.273 | -0.7587 | Yes |
| 43 | HMGCR |  |  | 9640 | -0.277 | -0.7502 | Yes |
| 44 | CCDC47 |  |  | 9664 | -0.291 | -0.7430 | Yes |
| 45 | PDIA3 |  |  | 9687 | -0.301 | -0.7354 | Yes |
| 46 | LRPAP1 |  |  | 9691 | -0.305 | -0.7256 | Yes |
| 47 | HACD3 |  |  | 9697 | -0.311 | -0.7159 | Yes |
| 48 | PDIA6 |  |  | 9701 | -0.314 | -0.7058 | Yes |
| 49 | DSEL |  |  | 9714 | -0.327 | -0.6963 | Yes |
| 50 | TMBIM6 |  |  | 9716 | -0.328 | -0.6856 | Yes |
| 51 | MMP16 |  |  | 9724 | -0.335 | -0.6752 | Yes |
| 52 | APLP2 |  |  | 9736 | -0.344 | -0.6650 | Yes |
| 53 | SPCS2 |  |  | 9744 | -0.353 | -0.6541 | Yes |
| 54 | GPC1 |  |  | 9751 | -0.356 | -0.6429 | Yes |
| 55 | FUS |  |  | 9779 | -0.389 | -0.6329 | Yes |
| 56 | ATP6AP2 |  |  | 9783 | -0.396 | -0.6201 | Yes |
| 57 | MTRNR2L12 |  |  | 9787 | -0.402 | -0.6072 | Yes |
| 58 | SQLE |  |  | 9790 | -0.408 | -0.5939 | Yes |
| 59 | B2M |  |  | 9791 | -0.408 | -0.5805 | Yes |
| 60 | RTN3 |  |  | 9792 | -0.413 | -0.5668 | Yes |
| 61 | LPL |  |  | 9802 | -0.428 | -0.5536 | Yes |
| 62 | CANX |  |  | 9804 | -0.431 | -0.5395 | Yes |
| 63 | WLS |  |  | 9809 | -0.451 | -0.5251 | Yes |
| 64 | ABHD2 |  |  | 9812 | -0.476 | -0.5096 | Yes |
| 65 | VGF |  |  | 9816 | -0.487 | -0.4939 | Yes |
| 66 | ALCAM |  |  | 9818 | -0.493 | -0.4777 | Yes |
| 67 | APP |  |  | 9819 | -0.495 | -0.4614 | Yes |
| 68 | GPM6A |  |  | 9820 | -0.499 | -0.4449 | Yes |
| 69 | PGRMC1 |  |  | 9826 | -0.516 | -0.4284 | Yes |
| 70 | SLC44A1 |  |  | 9831 | -0.527 | -0.4114 | Yes |
| 71 | CD9 |  |  | 9832 | -0.544 | -0.3935 | Yes |
| 72 | PLAT |  |  | 9833 | -0.550 | -0.3754 | Yes |
| 73 | PON2 |  |  | 9835 | -0.554 | -0.3572 | Yes |
| 74 | GPNMB |  |  | 9839 | -0.577 | -0.3385 | Yes |
| 75 | LAPTM4B |  |  | 9840 | -0.579 | -0.3194 | Yes |
| 76 | MDM4 |  |  | 9841 | -0.601 | -0.2996 | Yes |
| 77 | GRIA2 |  |  | 9846 | -0.638 | -0.2790 | Yes |
| 78 | FGFBP3 |  |  | 9850 | -0.651 | -0.2578 | Yes |
| 79 | KCNQ1OT1 |  |  | 9861 | -0.751 | -0.2341 | Yes |
| 80 | NCAM1 |  |  | 9862 | -0.751 | -0.2093 | Yes |
| 81 | SOX6 |  |  | 9863 | -0.798 | -0.1830 | Yes |
| 82 | MALAT1 |  |  | 9868 | -0.912 | -0.1533 | Yes |
| 83 | PTPRZ1 |  |  | 9869 | -0.918 | -0.1231 | Yes |
| 84 | PTN |  |  | 9874 | -1.107 | -0.0870 | Yes |
| 85 | BCAN |  |  | 9878 | -1.175 | -0.0486 | Yes |
| 86 | SCRG1 |  |  | 9879 | -1.473 | 0.0000 | Yes |
Table: GSEA details [plain text format]

  

Fig 2: LARSSON\_STATE\_3: Random ES distribution      
 Gene set null distribution of ES for **LARSSON\_STATE\_3**
